# Uncertainty Quantification for Epidemic Risk Management: Case of SARS-CoV-2 in Morocco

**DOI:** 10.3390/ijerph20054102

**Published:** 2023-02-24

**Authors:** Lamia Hammadi, Hajar Raillani, Babacar Mbaye Ndiaye, Badria Aggoug, Abdessamad El Ballouti, Said Jidane, Lahcen Belyamani, Eduardo Souza de Cursi

**Affiliations:** 1 Laboratory of Engineering Sciences for Energy, National School of Applied Sciences ENSAJ, UCD, El Jadida 24000, Morocco; 2 Laboratory of Mechanics of Normandy, National Institute of Applied Sciences INSA of Rouen-Normandy, 76800 Saint Etienne du Rouvray, France; 3 Laboratory of Mathematics of Decision and Numerical Analysis, University of Cheikh Anta Diop, Dakar 10700, Senegal; 4 Emergency Department, SAMU 02, CHU Ibn Rochd, Casablanca 20100, Morocco; 5 Emergency Department, Mohammed V Military Hospital, Faculty of Medicine and Pharmacy, Mohammed V University, Rabat 10100, Morocco

**Keywords:** uncertainty quantification, Hilbert expansion, risk management, epidemic risk, SARS-CoV-2, moment matching, collocation

## Abstract

In this paper, we propose a new method for epidemic risk modelling and prediction, based on uncertainty quantification (UQ) approaches. In UQ, we consider the state variables as members of a convenient separable Hilbert space, and we look for their representation in finite dimensional subspaces generated by truncations of a suitable Hilbert basis. The coefficients of the finite expansion can be determined by approaches established in the literature, adapted to the determination of the probability distribution of epidemic risk variables. Here, we consider two approaches: collocation (COL) and moment matching (MM). Both are applied to the case of SARS-CoV-2 in Morocco, as an epidemic risk example. For all the epidemic risk indicators computed in this study (number of detections, number of deaths, number of new cases, predictions and human impact probabilities), the proposed models were able to estimate the values of the state variables with precision, i.e., with very low root mean square errors (RMSE) between predicted values and observed ones. Finally, the proposed approaches are used to generate a decision-making tool for future epidemic risk management, or, more generally, a quantitative disaster management approach in the humanitarian supply chain.

## 1. Introduction

In Morocco, the number of cumulative confirmed cases of SARS-CoV-2 is currently 1,258,761 with 7209 individuals undergoing treatment in July 2022 [1,2]. The first cases, from Wuhan, were notified to the WHO on 31 December 2019 [3,4] while, Morocco notified its first case on 02 March 2020 [1]. As in many countries of the world, it is valuable to understand the growth and the timing in responding to the logistic needs of the health system [5,6,7]. The aim of the present paper is to study different wave comparisons of the number of virus patients, visualization of the most critical distribution of contamination cases in Morocco and the cumulative distribution function of the number of confirmed cases and deaths.

As an approach, we use a mathematical model and an uncertainty quantification technique for epidemics that integrates the different stages of individuals (susceptible, deaths, recovered, exposed and asymptomatic). We consider that our analysis highlights the importance of anticipation and timing to avoid overwhelming in the health system [7,8,9]. More precisely, our analysis shows, in particular, that, while the epidemic growth is exponentially fast, by respecting a certain logistic growth, the epidemic might remain under control [6]. As a consequence, a non-negligible number of expenses can be saved. Finally, a probative measure approach to project forwards the cumulative number of virus-infected cases is provided.

Let us mention that several studies have been conducted in different contexts to assess the impact of COVID-19 on hospital saturation, economy, clinical practice, and in human and environmental terms. We refer for examples to [7,8,9,10,11,12,13,14,15] and the references therein. Our work complements several studies that have been carried out on the spread of SARS-CoV-2 in Africa [16,17,18,19,20,21,22,23,24].

Epidemic risk modeling and prediction is not new [25]. However, we aim to take into account the sources of uncertainties in the model and to test their influence on the accuracy of the results. The uncertainties can be of an aleatory or epistemic type, and can have different sources, namely, input data, model bias or numerical errors [26]. Uncertainties cannot be avoided, so, the effective solution is to quantify them by calibrating the proposed models and by using these calibrated models for making predictions, estimations and/or approximations. SARS-CoV-2 modelling is fundamentally uncertain: on the one hand, for the asymptomatic cases, the available data are limited and sometimes based on approximations. On the other hand, the algorithms used for modelling are themselves uncertain: they are only an approximation of reality and include many unknowns (speed of propagation, virus mutation, etc.) They do not give enough information for the estimation of possible impacts and damages. All these uncertainties significantly influence the results of the modelling and must be considered.

Uncertainty quantification has gained massive attention from many researchers in different fields, namely, applied mathematics, engineering, physics, management and economics. Uncertainty quantification in terms of state estimation of discrete/continuous time systems is a fundamental problem in engineering sciences [27]. UQ can be defined as that process of quantifying all sources of uncertainties linked to the systems and testing their influence on the accuracy of the results in order to: (i) keep their desired behavior, (ii) avoid undesired states, faults and damages, (iii) to furnish a realistic prognosis in the future, and so on. UQ tools has been used in several studies to assess and manage risks. Hammadi et al., 2022 [27,28] suggested a model using uncertainty quantification for risks linked to the customs supply chain based on the moment matching method and considering seasonality of illicit traffics in five sites in Morocco. Lopez et al., 2017 [29] has used methods of uncertainty quantification for differential equations in order to predict and model the propagation of influenza in boarding school using three approaches: moment matching (MM), collocation (COL) and variational (VAR), including a discrete linear system. Abdo et al., 2017 [30] developed a model based on uncertainty quantification for atmospheric dispersion risk assessment and treatment by using five methods of uncertainty representation and characterization, namely: probability theory, interval analysis, the fuzzy approach, the mixed probabilistic–fuzzy approach and the evidence theory. In our application, the aim is to develop an uncertainty quantification model for epidemic risk, taking into consideration the uncertainties linked to input data. Based on the fitted models, both a determination of the cumulative distribution function and a prediction of the most significant indicators of SARS-CoV-2 in Morocco are provided.

The rest of the paper is organized as follows: a brief introduction which recalls the general framework of uncertainty quantification and fixes the notation employed in this text is given in Section 2. Section 3 presents the two methods for the numerical determination of the coefficients of the expansion with examples in order to validate the proposed approach. It is followed by Section 4, concerning the estimation of the unknown parameters of the model and a discussion of numerical results. In Section 5, we propose a new approach for managing future epidemic risks. Finally, Section 6 presents the main conclusion and future works drawn from this work.

## 2. Uncertainty Quantification-Based Model

In this section, we introduce the theoretical framework of uncertainty quantification and the parameter setting of our proposed model for epidemic risk.

### 2.1. Essence of Uncertainty Quantification

Uncertainty quantification (UQ) is a practice for making a model-based prediction, estimation and/or approximation reliable. Uncertainties can be of different types: stochastic (aleatory) or epistemic. Stochastic uncertainties that come from the inherent randomness of natural phenomena can be generated by either sampling uncertainty or measurement uncertainty [26]. Epistemic uncertainties are those generated from the lack of information or knowledge about the phenomena; they may have different sources, namely: data, parameters, algorithmic or method uncertainty [26]. 

Many studies were conducted to handle the uncertainties and researchers developed various methods in order to improve the quality and the efficiency of UQ (see, for instance, [31,32,33,34]). Among these methods, Monte Carlo simulation (MCS) may be considered as being the basic UQ approach to be used when no other information is known. The MCS provides successive resolutions of a deterministic system incorporating uncertain parameters modeled by random variables. Historically, only the generation of variables is available, and this technique was developed for these types of situations with a lack of information. It generates, for uncertain parameters and according to their probability distributions and their correlations, random draws. For each draw, a set of parameters is obtained and a deterministic calculation, following well defined analytical or numerical models, is made. The statistics generated by this method are poor in accuracy [35] and significant results require a large number of samples which makes the MCS prohibitive in terms of computational cost [36]. Improvements may be obtained by using Latin hypercube sampling (LHS). Such a sampling approach gives a better accuracy [35]. However, it has some limitations because even if the number of samples increases, the error estimates may not be enhanced [28].

To avoid this kind of problem, polynomial chaos expansion (PCE) [37] was introduced. The original PCE was based on the representation of random variables by a multivariate series of polynomials of Gaussian variables [28,38,39]. Nowadays, it has evolved to consider an arbitrary Hilbert basis and other distributions (see Examples in Section 3). Although, PCE has proved to be less expensive and computationally more accurate than the MCS and LHS, this approach needs previous knowledge of the distributions considered, which generate some difficulties, so that variations were introduced, tending to use samples instead of the random variables themselves.

### 2.2. The Model Situation

Uncertainty quantification models are oriented towards the determination of probability distributions (cumulative or density functions) of random variables in its general model. UQ considers a system having entries $X\in {\mathbb{R}}^{k_{X}}$, internal parameters $U\in {\mathbb{R}}^{k_{U}}$ and an output $Z\in {\mathbb{R}}^{k_{Z}}$: Z is the system’s response to the entry X, and the system is characterized by its parameters U. Both the parameters and the entry can be affected by uncertainty, so that they are grouped in a vector of uncertain variables $S=\left(X,U\right)\in {\mathbb{R}}^{k_{S}}$, $k_{S}=k_{X}+k_{U}$. In the UQ framework, ***Z*** is a function of ***S***, ***Z*** = ***Z*** (***S***) and some statistical information is available on the couple (S,Z)—for instance, a finite sample of the couple. UQ also proposes methods for the situation where ***S*** remains totally or partially unobserved and only observations of ***Z*** are available, by introducing a convenient artificial random vector T, destined to replace the unobserved parts of S with observed ones. 

The Hilbert approach in the UQ framework considers a probability space (Ω, P) and the separable Hilbert space $H=L^{2}\left(\Omega ,\;P\right)$ with scalar product ${\left(X,Y\right)}_{H}=E\left(XY\right)$. Let $S\in H^{k_{S}}$ be such that $Im\left(S\right)=I\subset {\left(a,b\right)}^{k_{S}}\subset {\mathbb{R}}^{k_{S}}$ and $Z:I\to \;{\mathbb{R}}^{k_{Z}}$ be such that Z=Z(S). Under suitable conditions [40], we can consider a Hilbert basis $ℬ={\left\{\varphi_{i}\right\}}_{i\in {\mathbb{N}}^{*}}\subset L^{2}\left(a,b\right)$ (${\mathbb{N}}^{*}$ is the set of the strictly positive integers) and represent each component of $Z=\left(Z_{1},\;\ldots ,\;Z_{k_{Z}}\right)$ as an expansion [28,36,41,42]:

(1) $$Z_{j}=\underset{i\in {\mathbb{N}}^{*}}{\sum }z_{ij}\varphi_{i}\left(S\right)\;\;\;\;\;,\;z_{ij}\in \mathbb{R}\;,\;1\leq j\leq k_{Z}$$

Notice that the random variables ***Z*** and S are defined on the probability space (Ω,P), while the Hilbert basis ℬ is not probabilistic and takes real vectors as arguments and produces real vectors as results. Such a choice is possible since the image of S is formed by real vectors. The literature proposes methods for the generation of a Hilbert basis by considering eigenfunctions of convenient operators—see, for instance [43]. Most of the bases proposed in the literature are polynomial or trigonometrical. In this work, we shall use a polynomial basis.

The expansion (1) can be written in vectorial form as

(2) $$Z=\underset{i\in {\mathbb{N}}^{*}}{\sum }z_{i}\varphi_{i}\left(S\right)\;\;,\;z_{i}=\left(z_{i1},\ldots ,z_{ik_{Z}}\right)\in {\mathbb{R}}^{k_{Z}}$$

In practice, a truncation of the series is used:

(3) $$Z\approx P_{n}Z=\overset{n}{\underset{J=1}{\sum }}z_{J}\varphi_{j}\left(S\right)$$

Examples of Hilbert bases can be found in the literature, namely, polynomial and trigonometrical bases. We can find works in the literature involving non orthogonal, orthogonal, or orthonormal bases. In this work, we shall consider a non-orthogonal polynomial basis: $P_{n}Z$ is a polynomial function of S.

The coefficients z can be assembled in a matrix:

(4) $$\;𝕫=\left(z_{ij}\;:1\leq i\leq n,1\leq j\leq k_{Z}\right)\in {\mathbb{R}}^{n\times k_{Z}},$$

and we have

(5) $$Z=\Phi \left(S\right)𝕫\;,\;\;\;\Phi \left(S\right)=\left(\varphi_{1}\left(S\right),\;\ldots ,\varphi_{n}\left(S\right)\right)$$

$P_{n}Z$ corresponds to an orthogonal projection: let $V_{n}$ be the linear subspace of $H^{k_{S}}$ given by

(6) $$\;V_{n}=\left\{Y\in H^{k_{S}}\;:\;Y=\Phi \left(S\right)𝕪\;:\;𝕪\in {\mathbb{R}}^{n\times k_{Z}}\right\}$$

Notice that $V_{n}={\left[{ℬ}_{n}\right]}^{k_{Z}}$, where $\left[{ℬ}_{n}\right]$ is the linear span of ${ℬ}_{n}=\left\{\varphi_{1}\left(S\right),\;\ldots ,\varphi_{n}\left(S\right)\right\}$. The best approximation of Z in $V_{n}$ is its orthogonal projection onto $V_{n}$, denoted as $P_{n}Z$. We have $P_{n}Z\to Z$ for n→+∞.

The coefficients 𝕫 must be determined by appropriated methods—for instance, by collocation or moment matching. The literature shows that such an approach allows the calculation of probabilities of events related to **Z** by using, the distribution of **PZ**, with good results [44].

In the framework of the Hilbert space ${\left[L^{2}\left(\Omega ,\;P\right)\right]}^{k_{Z}}$, the determination of the orthogonal projection $P_{n}Z$ involves the solution of the variational equation

(7) $$P_{n}Z=\Phi \left(S\right)𝕫\;\;\mathrm{and}\;{\left(Z-P_{n}Z,Y\right)}_{H^{k_{Z}}}=0,\forall Y\in V_{n}.$$

Taking Y=Φ(S)𝕪, we obtain

(8) $${𝕫}^{t}E\left(\Phi {\left(S\right)}^{t}\Phi \left(S\right)\right)𝕪=E\left(Z^{t}\Phi \left(S\right)\right)𝕪,\;\;\;\;\forall 𝕪\in {\mathbb{R}}^{n\times k_{Z}},\;$$

so that

(9) $${𝕫}^{t}E\left(\Phi {\left(S\right)}^{t}\Phi \left(S\right)\right)=E\left(Z^{t}\Phi \left(S\right)\right)$$

Equation (9) can be transformed in a linear system or a family of $k_{Z}$ linear systems [32,44,45], but the determination of 𝕫 by this method involves the determination of $E\left(\varphi_{i}\left(S\right)\varphi_{j}\left(S\right)\right)$, $E\left(Z_{\ell }\varphi_{j}\left(S\right)\right)$ for 1≤i,j≤n, $1\leq \ell \leq k_{Z}$, so that it requires knowledge of the joint distribution of the pair (S,Z). Such information can be unavailable, so that alternative approaches, based on samples, were developed [36,41,42].

If Z depends on time t, id est, Z=Z(t), the coefficients 𝕫 become functions of time ${𝕫}_{ij}={𝕫}_{ij}\left(t\right),\;1\leq i\leq n,\;1\leq j\leq k_{Z}$ [36,41,42]. In our application:

S is a random vector representing the aleatory/epistemic uncertainties in the health system, and

Z denotes the daily number of infected people and deaths by SARS-CoV-2 in Morocco.

## 3. Numerical Methods for UQ

In this section, we present two *non-intrusive* methods for the evaluation of the coefficients $𝕫\in {\mathbb{R}}^{n\times k_{Z}}$ defining the approximation $P_{n}Z$. 

Indeed, we can find in the literature methods for their determination, which are usually classified in *intrusive* or *non-intrusive*. 

Intrusive approaches request a numerical or theoretical model, which is modified: the coefficients are introduced in the model by replacing Z with $P_{n}Z$ and generating modified equations by adapted methods, such as using orthogonal projections. As an example, let us consider a model defined by a vectorial system of algebraic equations ψ(S,Z)=0: we rewrite this equation as $E\left(\varphi_{i}\left(S\right)\psi \left(S,P_{n}Z\right)\right)=0,\;i=1,\;\ldots n$, which generates a system of nonlinear equations for the coefficients z**.** To exemplify, we can consider the simple model ψ(S,Z)=S−Z=0. In this case, we obtain Equation (6), which is linear. In a general situation, we obtain a nonlinear system, which must be solved by a convenient method to determine z. Such an approach is intrusive since it is necessary to intervene in the model. 

Non-intrusive methods do not require model modifications—they can even be used when the model is not available, as they are most often based on samples. For instance, non-intrusive approaches can be used when ψ is unknown, but we have a sample $S=\left\{\left(S_{i},Z_{i}\right):1\leq i\leq m_{s}\right\}$. Examples of non-intrusive approaches are those furnished by moment matching (MM) and collocation (COL), which are used in this work. 

In the next subsections, we are going to briefly present MM and COL, including simple examples destined to clarify for the reader the steps requested for their implementation, namely, the choice of the random variable S.

We present the results of this section as the cumulative density function (CDF) of the state variables ***Z***. From the CDF, one may obtain any desired statistical information of the state variables, such as mean value, variance, or any given quantile of interest. If desired, the probability density function (PDF) can be determined by numerical derivation of the CDF—in this task, particle derivatives have been shown to be efficient [41,42,46]. In this work, PDF is not requested, since we are mainly interested in statistical quantities and probabilities that can be determined from the CDF.

### 3.1. Approach by Collocation (COL)

Fitting the underlying model, for given a distribution of the couple (***S***, ***Z***), consists of determining the coefficients 𝕫 of the approximation. As previously observed, these coefficients are defined by a variational equation, which can be reduced to a linear system, but whose solution requests the knowledge of the joint distribution of the pair (***S***, ***Z***). Thus, in practice, it may be necessary to use an approach based on a sample $S=\left\{\left(S_{i},Z_{i}\right):1\leq i\leq m_{s}\right\}$. COL is such an approach: it is an interpolation method, equivalent to the approximation of the means in Equation (9) by their estimations on the sample [42]. In COL, we consider that the approximation coincides with the real value on the sample:

(10) $$P_{n}Z\left(S_{i}\right)=Z_{i},\;\;1\leq i\leq m_{s}$$

Then,

(11) $$\Phi \left(S_{i}\right)𝕫=Z_{i}$$

Let us assemble $Z_{1},\ldots .,\;Z_{m_{s}}$ into a matrix ℤ having as line i the vector formed by the values of $Z_{i}$: for $Z_{i}=\left({\left(Z_{i}\right)}_{1},\ldots {\left(Z_{i}\right)}_{k_{Z}}\right)\in {\mathbb{R}}_{Z}^{k}$, we have ${\mathbb{Z}}_{ij}={\left(Z_{i}\right)}_{j}$. Then 𝕫 is the solution of a linear system:

(12)   F𝕫=G, 

with $F\in {\mathbb{R}}^{m_{s}\times n},\;G\in {\mathbb{R}}^{m_{s}\times k_{Z}}$, given by

(13) $$\;F_{ij}=\varphi_{j}\left(S_{i}\right)\;\;,\;G_{i\ell }={\mathbb{Z}}_{i\ell }\;\;,\;1\leq i\leq m_{s},\;1\leq j\leq n,\;1\leq \ell \leq k_{Z}$$

This linear system involves $m_{s}\times k_{Z}$ equations for $n\times k_{Z}$ unknowns. For: $m_{s}>n$, the number of equations is higher than the number of unknowns and the system is overdetermined: it must be solved by a generalized solution, such as, for instance, the minimum squares one. As pointed out previously, the minimum squares solution corresponds to the approximation of the means in Equation (9) by their estimations on the sample S. It can also be interpreted as a discrete version of the Hilbertian approximations on the finite dimensional subspaces $V_{n}$ [36]. Other interpolation methods can be considered, such as collocation by intervals method or spline approximations.

As a first example, let us consider a situation where $k_{Z}=k_{S}=2$, $Z=\left(S_{1}+S_{2},S_{1}S_{2}\right)$, with $S_{1}∼N\left(0,1\right)$; $S_{2}∼Exp\left(1\right)$; $S_{1},\;S_{2}$ independent. We consider a sample of 100 variates from (S,Z), generated by $\left(S_{1}{}_{i},\;S_{2}{}_{j}\right),\;1\leq i,j\leq 10$ (Table 1).

We use a polynomial basis given by

(14) $$\;\varphi_{k}\left(S\right)=\varphi_{1,i}\left(S_{1}\right)\varphi_{2,j}\left(S_{2}\right),\;\;k=\left(i-1\right)k_{2}+j\;\;,\;1\leq i\leq k_{1},\;1\leq j\leq k_{2}$$

with

(15) $$\varphi_{i,\ell }\left(S\right)={\left(\frac{S_{i}-a_{i}}{b_{i}-a_{i}}\right)}^{\ell -1},\;a_{i}=\min\left({S_{i}}_{p}\right),\;b_{i}=\max\left({S_{i}}_{p}\right),\;1\leq p\leq 10$$

As previously explained, this choice is suggested by the fact that many Hilbert bases considered in the literature are polynomial. The index transformation used is classical in the framework of finite element methods and uncertainty quantification (see, for instance, [44,45]) and allows the determination of the coefficients by standard methods for the solution of linear systems of algebraic equations.

**Figure 1:**
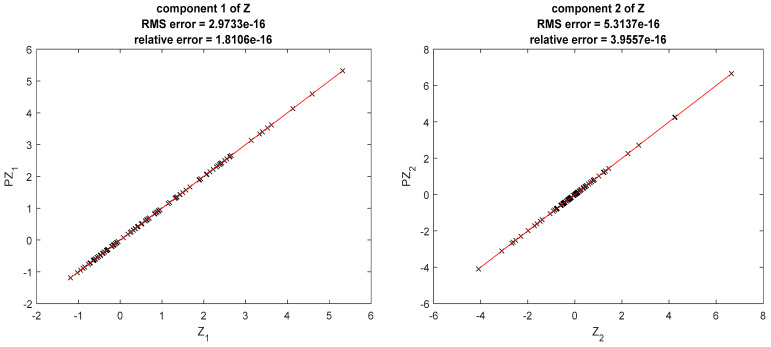
Results obtained in the first example. Points marked on the graphics correspond to $\left(Z_{i}\left(S_{k}\right),PZ_{i}\left(S_{k}\right)\right)$. Close results correspond to points close to the bisector of the quadrant. RMS errors. are inferior to 1 × 10^−15^.

The results obtained with $k_{1}=k_{2}=3$ are shown in Figure 1.

We compared the prediction furnished by $P_{n}Z$ on the data, on an uniform grid of 201×201 points on $\left(a_{1},\;b_{1}\right)\times \left(a_{2},b_{2}\right)$ and on a random sample of 217×235 points on $\left(a_{1},\;b_{1}\right)\times \left(a_{2},b_{2}\right)$ (we generated 250 variates of $S_{i},\;i=1,2$ with the normal and the exponential generators from Matlab. Points outside the region were excluded from the sample). The errors are synthetized in Table 2.

**Figure 2:**
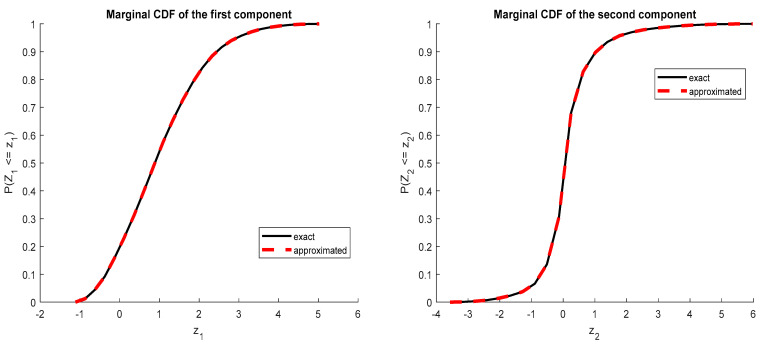
Cumulative density function obtained by collocation in the first example.

The marginal distributions were determined and are shown in Figure 2.

**Figure 3:**
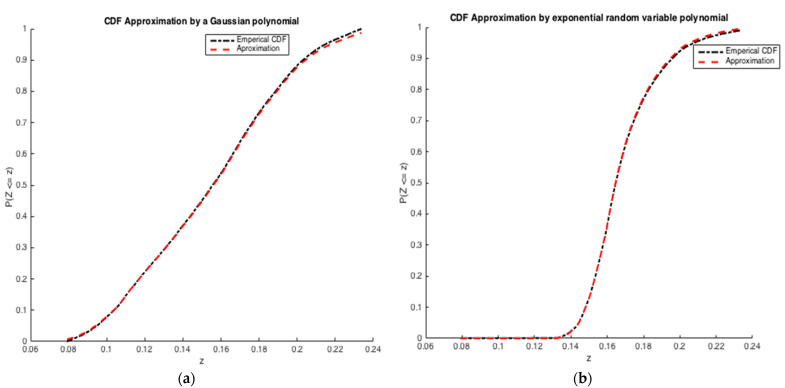
Exponential variable approximated using collocation by: (**a**) a Gaussian polynomial and, (**b**) an exponential random variable polynomial.

As a second example, let us consider $k_{Z}=k_{S}=1$, with Z normally distributed, $Z∼N\left(m_{Z}=0.052,\;\sigma_{Z}^{2}=0.011\right)$. We assume that a sample of 100 variates of Z is available, but S is unknown. We consider two artificial choices for S: (i) a Gaussian variable N(0,1) (independent from Z), (ii) an exponential random variable Exp(1). Using n=7 and $\varphi_{i}$ given by Equation (15), we obtain the coefficients in Table 3:

The cumulative distribution function associated with 𝐏𝐙 is compared to the exact one in Figure 3, as we see, the approximation has a good quality in the two cases.

### 3.2. Approach by Moment Matching (MM)

Moment matching looks for an element $P_{n}Z$ such that a finite set of its moments coincides with those of Z. In practice, a sample is used to evaluate the moments [27,36,41,42]. 

Let us consider a multi-index $\ell =\left(\ell_{1},\ldots ,\ell_{k_{Z}}\right)\in {\left({\mathbb{N}}^{*}\right)}^{k_{Z}}$ and denote $\left|\ell \right|=\ell_{1}+\cdots +\ell_{k_{Z}}$. We consider $ℐ\left(k\right)=\left\{\ell \in {\left({\mathbb{N}}^{*}\right)}^{k_{Z}}\;:\;\left|\ell \right|\leq k\right\}$ and we set, for $X=\left(X_{1},\;\ldots ,X_{k_{Z}}\right)$ and ℓ∈ℐ(k):

(16) $$M_{\ell }\left(X\right)=E\left(\overset{k_{Z}\;}{\underset{i=1}{\prod }}X_{i}^{\ell_{i}}\;\right).$$

Moment matching looks for an approximation $P_{n}Z$ such that

(17) $$M_{\ell }\left(Z\right)=M_{\ell }\left(P_{n}Z\right),\;for\;\ell \in ℐ\left(k\right).$$

Let us consider a $F_{\ell }:{\mathbb{R}}^{n\times k_{Z}}\to \;\mathbb{R}$ given by

(18) $$F_{\ell }\left(𝕫\right)=M_{\ell }\left(\Phi \left(S\right)𝕫\right).$$

Then, the coefficients 𝕫 correspond to the solution of the nonlinear algebraic system

(19) $$𝕫\in {\mathbb{R}}^{n\times k_{Z}}\;\;\mathrm{and}\;\;F_{\ell }\left(𝕫\right)-M_{\ell }\left(Z\right)=0,\;\forall \ell \in ℐ\left(k\right).$$

We can also consider the solution of

(20) $$\;𝕫\in {\mathbb{R}}^{n\times k_{Z}}\;\;\mathrm{and}\;\frac{\;F_{\ell }\left(𝕫\right)-M_{\ell }\left(Z\right)}{M_{\ell }\left(Z\right)}=0,\;\forall \ell \in ℐ\left(k\right).$$

Numerically, we can look for

(21) $$𝕫=\arg\min\left\{F\left(𝕪\right)=\underset{\ell \in ℐ\left(k\right)}{\sum }d\left(F_{\ell }\left(𝕪\right),M_{\ell }\left(Z\right)\right):𝕪\in {\mathbb{R}}^{n\times k_{Z}}\;\right\},$$

where d:ℝ×ℝ→ ℝ verifies d(x,y)≥0, d(x,y)=0 ⇔ x=y. Examples of possible choices for d are:

(22) $$d_{1}\left(x,y\right)=\left|x-y\right|\;\;;d_{2}\left(x,y\right)={\left|x-y\right|}^{2},\;d_{1,rel}\left(x,y\right)=\frac{\left|x-y\right|}{\left|y\right|}$$

When $k_{Z}=1$, the equations simplify: we have

(23) $$𝕫\in {\mathbb{R}}^{n}\;\;\mathrm{and}\;\;F_{\ell }\left(𝕫\right)=M_{\ell }\left(Z\right),\;\;1\leq \ell \leq k.$$

or

(24) $$\;\;𝕫\in {\mathbb{R}}^{n}\;\;\mathrm{and}\;\;\frac{\;F_{\ell }\left(𝕫\right)-M_{\ell }\left(Z\right)}{M_{\ell }\left(Z\right)}=0,\;\;\;\;\;1\leq \ell \leq k.$$

and

(25) $$\;𝕫=\arg\min\left\{F\left(𝕪\right)=\overset{k}{\underset{\ell =1}{\sum }}d\left(F_{\ell }\left(𝕪\right),M_{\ell }\left(Z\right)\right):𝕪\in {\mathbb{R}}^{n}\;\right\},$$

The main difficulty in moment matching is the numerical solution of Equations (19) and (20) or (21) (or (23), (24), (25) for $k_{Z}=1$). Indeed, the equations are strongly nonlinear and 𝕪→F(𝕪) is nonconvex, so that a global optimization algorithm must be employed. In addition, MM is based on Levy’s theorem [36,41,42].

Theorem (Levy): Let $\left\{X_{n}\right\}$ be a sequence of random variables and $\phi_{n}\left(t\right)=E\left(e^{itX_{n}}\right)$ be the characteristic function of $X_{n}$. Let X be a random variable and $\phi \left(t\right)=E\left(e^{itX}\right)$ be its characteristic function. Then $X_{n}\to X$ in distribution if and only if $\phi_{n}\left(t\right)\to \phi \left(t\right)$ almost everywhere.

Notice that this theorem ensures only convergence in distribution, so that the variable $P_{n}Z$ may not converge to **Z**.

**Figure 4:**
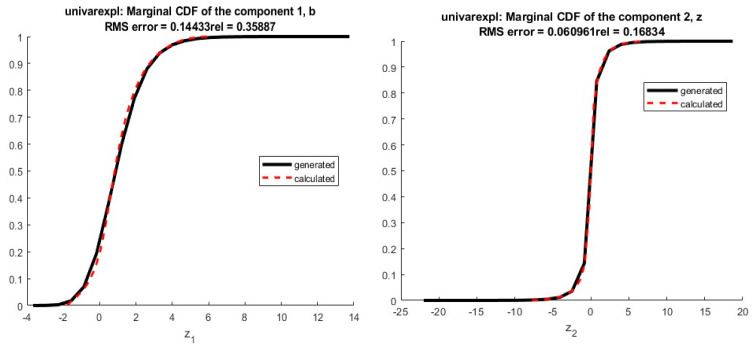
Cumulative density function obtained by MM in the first example.

As a first example, let us consider again the situation where $k_{Z}=k_{S}=2$, $Z=\left(S_{1}+S_{2},S_{1}S_{2}\right)$, with $S_{1}∼N\left(0,1\right)$; $S_{2}∼Exp\left(1\right)$; $S_{1},\;S_{2}$ independent. Assume that we do not have any information about S: only the values of Z are available. In such a situation, we do not know how many random variables must be used to describe the data and we do not know their distributions. This lack of information can be completed by using more data: assume that we have a sample formed by 400 variates of Z and let us use moment matching: we shall try to represent each component of Z by using a single random Gaussian variable, id est, that $Z_{i}=Z_{i}\left(S_{i}\right)$, where $S_{i}∼N\left(0,1\right)$. To do this, we generate two independent samples of 400 variates from $S_{1}$ and $S_{2}$. Then we look for a representation of $Z_{i}$ as a polynomial of $S_{i}$ using moment matching. We considered 4 alternative methods of solution: (23), solving (24), (25) with $d_{1}$, (25), with $d_{1,rel}$. We used n=6 and tested k∈{8,9,…, 12}. All these choices led to results analogous to those shown in Figure 4—the optimization was carried by fminsearch (Matlab) with stochastic perturbations introduced via an output function, the results shown correspond to k=8. The coefficients found are shown in Table 4. 

The cumulative distribution function associated with 𝐏𝐙 is compared to the exact one in Figure 4. Again, the results are good.

**Figure 5:**
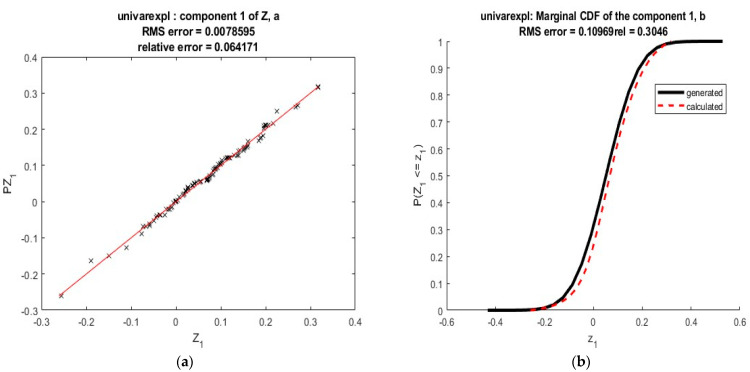
Gaussian variable approximated using moment matching: (**a**) polynomial expansion. (**b**) Cumulative density function. Results corresponding to a Gaussian polynomial.

As a second example, let us consider again ***Z*** normally distributed $∼N\left(m_{Z}=0.052,\;\sigma_{Z}^{2}=0.011\right)$. A sample of 100 variates is used to estimate the cumulative distribution function CDF based on the moment matching method as shown in Figure 5. An approximated solution is obtained by an expansion of degree n=6 with $\varphi_{i}\left(S\right)$ defined by (15) and S∼N(0,1). The coefficients obtained for k=12 are shown in Table 5. 

We observe that both the methods (collocation and moment matching) produce good results. Collocation requests less data, but more information about the distributions, which must be known. Moment matching requests more data, but produces results even for unknown distributions. In addition, the computational cost is much larger for MM, which requests the use of global optimization procedures, while collocation involves the solution of a linear system, generally of small or medium size. 

## 4. Numerical Analysis to SARS-CoV-2

Making the approximation and determining the cumulative distribution function (CDF) of an epidemic risk time series, we apply our approach by considering a polynomial basis ${\left\{\varphi_{i}\right\}}_{i\in {\mathbb{N}}^{*}}$, then we look for a finite expansion based on collocation and the moment matching method. In the following sections, we focus on the case of SARS-CoV-2 in Morocco.

### 4.1. Data Analysis

The simulations were carried out from data in [1], from 02 March 2020 to 15 August 2022. The numerical tests were performed by using Python with the Panda library, on a computer with the following characteristics: Intel(R) Xeon(R) Silver 4214R CPU@ 2.4 GHz, 64.0 Gb of RAM, using a Windows system. According to daily reports, we first analyze the data and perform some data preprocessing before simulations. We obtain various summary statistics (per day), by giving the mean, standard deviation, minimum and maximum values, and the quantiles of the data (see Table 6).

**Figure 6:**
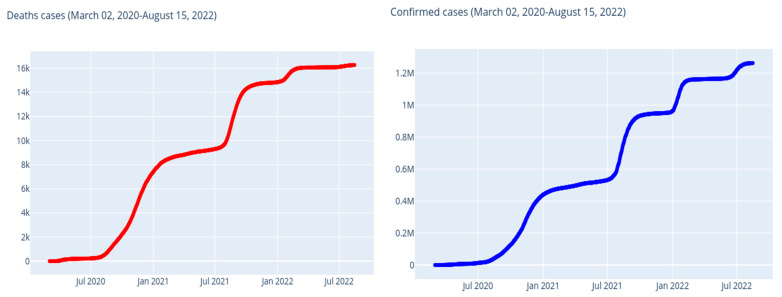
Morocco: evolution of the number of confirmed cases and deaths by SARS-CoV-2.

**Figure 7:**
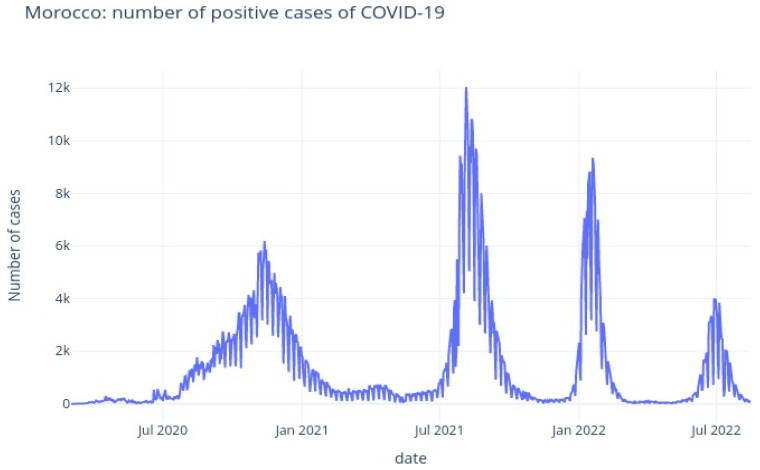
Morocco: evolution of cases per day.

Then, Figure 6 illustrates the cumulative numbers of confirmed and death cases, and Figure 7 illustrates the daily numbers of confirmed cases. The average increase in number of confirmed cases (each day) is 1403.0, and the average death count increase (every day) is 18.0.

**Figure 8:**
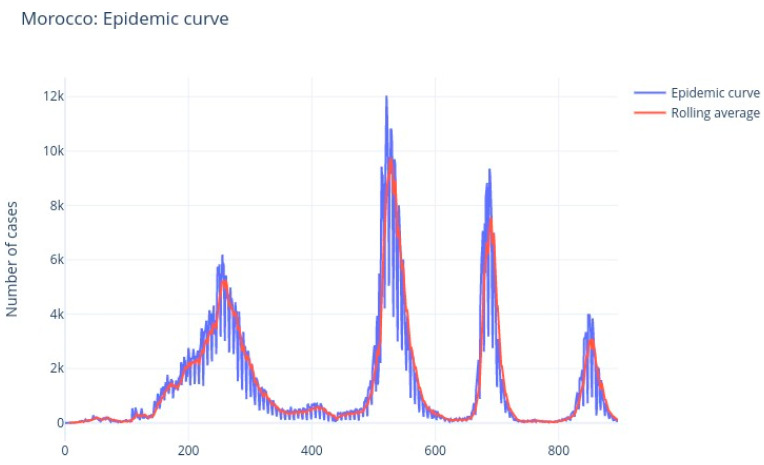
Morocco: COVID-19 epidemic curve.

**Figure 9:**
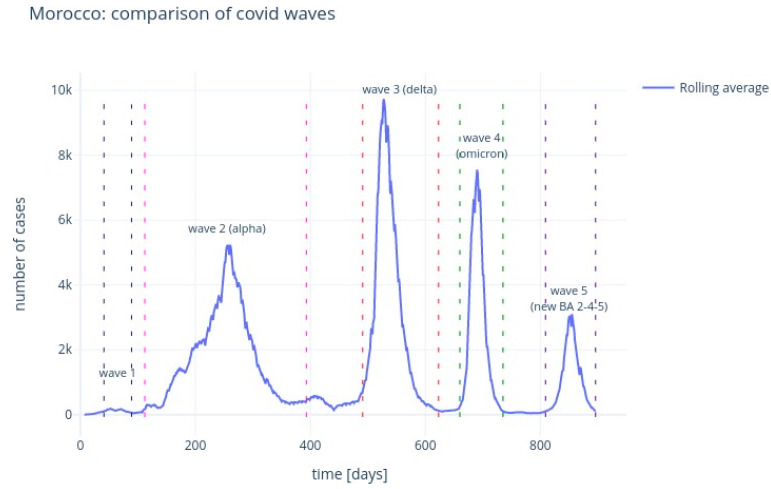
Morocco: COVID-19 five successive waves.

The Figure 8 and Figure 9 illustrate the rolling average of the five different waves, with:

- Small wave: from 11 April 2020 to 29 May 2020;
- Alpha wave: from 21 June 2020 to 29 March 2021;
- Delta wave: from 05 July 2021 to 14 November 2021;
- Omicron wave: from 21 December 2021 to 06 March 2022;
- BA 2-4-5 wave: from 19 May 2022 to 15 August 2022.

We see that wave 2 (alpha) lasted much longer (280 days). The largest peak was reached during wave 3 (delta), followed by omicron (wave 4), alpha (wave 2), waves 5 and 1.

In addition, let us define the fatality rate [19].

$$\mathrm{Fatality}\;\mathrm{rate}=\frac{\mathrm{number}\;\mathrm{of}\;\mathrm{death}\;\mathrm{cases}}{\mathrm{number}\;\mathrm{of}\;\mathrm{confirmed}\;\mathrm{cases}}\times 100$$

**Figure 10:**
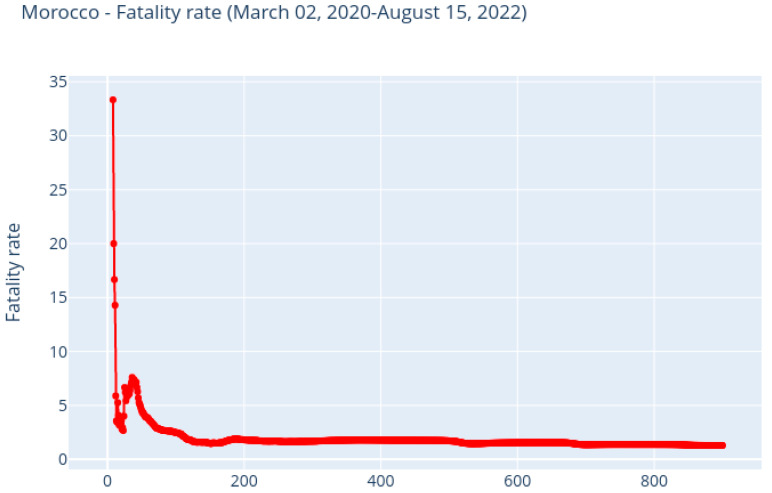
Morocco: The fatality rate.

The fatality rate is given by Figure 10, and its average value is 1.9268398. Due to the fact that the number of confirmed cases is much higher than the number of deaths, we can see that the fatality rate has started to fall again, which is a good sign.

### 4.2. Results and Discussion

In the following section, we present the numerical results of our proposed model based on UQ to analyze and predict the propagation of SARS-CoV-2 in Morocco. Based on the collected data in [1], from 02 March 2020 to 20 December 2021, we estimate main key pandemic parameters, especially CDFs, and make predictions on the infected and deaths to help with how to propose concrete actions to control the pandemic crisis. We highlight the importance of anticipation and timing to avoid overwhelming that could impact considerably the treatment of patients and the well-being of health care workers.

In the next subsections, the results are presented in the following manner: we first solve the state estimation problem using collocation and moment matching for the determination of the coefficients. Then, we calculate the CDFs of new infected cases and deaths as the main key pandemic parameters and test whether the epistemic uncertainties affect the results obtained. Also, we present the root mean square error (RMSE) results of the two approaches to compare their performance. Finally, we determine the probabilities related to humanitarian logistics which may be used to develop a new approach of risk management for potential epidemic risks faced in future in Morocco. The numerical tests were performed using Matlab [45]. The numerical experiments were executed on a computer intel(R) Core-i7 CPU 2.60 GHz, 24.0 Gb of RAM, using a UNIX system.

#### 4.2.1. Results Using Collocation

The effect of epidemic disease [44,47], such as SARS-CoV-2, is modeled here using a UQ model. It considers a fixed population with only three state variables: infected (I), recovered (R) and dead (D) people. “I” denotes the number of individuals who have been infected with the disease and are capable of spreading the disease to the susceptible category. “D” are those individuals who have been infected and then removed due to death. “R” represents individuals who have been infected and then removed due to treatment. Since we assume that the population is fixed, we only need to evaluate two of the three state variables, e.g., I and D.

**Figure 11:**
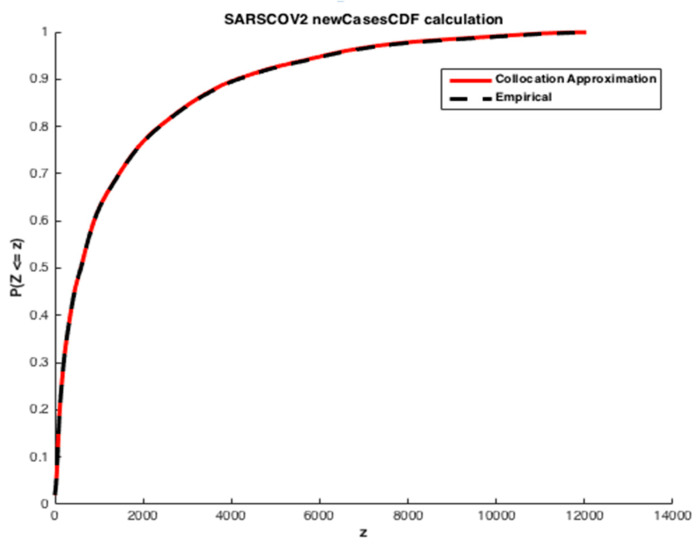
Estimation of the cumulative density function of the number of infected people by SARS-CoV-2 in Morocco. The results furnished by collocation.

**Figure 12:**
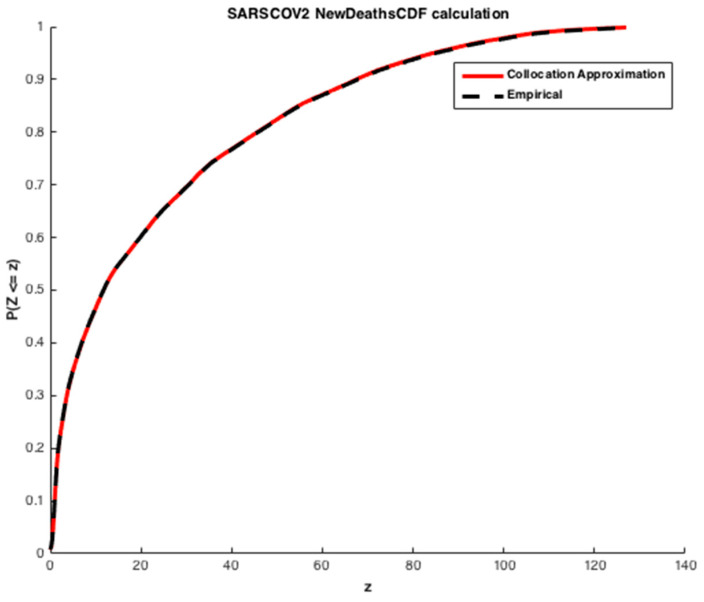
Estimation of the cumulative Density Function of the number of deaths by SARS-CoV-2 in Morocco. The results furnished by collocation.

We apply the collocation method with n=6 and $\varphi_{i}$ given by Equation (15), where S is exponentially distributed Exp(1). The cumulative distribution functions (CDFs) of the infected individuals and deaths are computed. The estimated values obtained are illustrated in Figure 11 and Figure 12.

We may see from the Figure 11 and Figure 12 that there is a good agreement between the estimated values of CDFs and the true response of the model. The coefficients of the orthogonal projection ***PZ*** are given in Table 7.

The orthogonal projection PZ approximated from the given data is as follow:

#### 4.2.2. Results Using Collocation Considering the Uncertainties in Data

**Figure 13:**
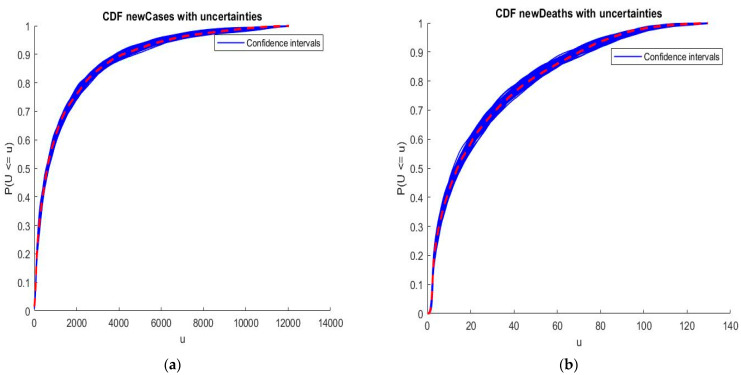
Estimation of the CDFs under uncertainties using Hausdorff distance: (**a**) CDF of infected individuals by SARS-CoV-2. (**b**) CDF of deaths by SARS-CoV-2.

Now, we suppose that there was a population not detected or asymptomatic, that was, however, affected by SARS-CoV-2. This population will be considered to be a random variable so that: $z^{\prime }=z+\Delta z$. The aim of this part is to validate the results of the previous section and test if the epistemic uncertainties, i.e., uncertainties in data, affect the results obtained by the collocation method. Uncertainties supposed to be in detections belong to the value range [0, 3] for both new cases and new deaths numbers. By using the Hausdorff distance we obtain the results in Figure 13. For that, we firstly review the computation of the Hausdorff distance [16]. Secondly, we apply this distance to compute the CDFs of key parameters of SARS-CoV-2 in Morocco. 

In mathematics, and more specifically in geometry, the Hausdorff distance is a topological tool that measures the distance between two subsets of an underlying metric space. This distance appears in two very different contexts: in the field of image processing and in mathematics. The Hausdorff distance consists in considering a metric space (W, d). Let A and D be two non-empty compact subsets of W. We first define, for any subset X of W, the open r-neighbourhood of X as being the set [48]:

(26) U (r, X)={x∈W|d (x, X)< r}

The Hausdorff distance $D_{h}$ (A, D) between A and D is defined as the smallest real number r so that the r-neighborhood of A contains D and the r-neighborhood of D contains A [16]. In other words:

(27) $$D_{h}\left(A,\;D\right)=\inf\;\left\{r>0|A\;∁\;U\left(r,\;D\right),\;D\;∁\;U\left(r,\;A\right)\right\}$$

The results presented in Figure 12 show that the uncertainties linked to non-detection of infected individuals or deaths by SARS-CoV-2 do not affect the results of CDFs computed by interpolation. Indeed, the CDFs under uncertainties have kept the same appearance of the distribution functions in Figure 11 and Figure 12. Consequently, the response of our proposed model of epidemic risk is not affected by epistemic uncertainties, i.e., those generated from the lack of information or knowledge about the phenomena, such as data, parameters, etc. So, the uncertainties that affected our study are of a stochastic type that come from the inherent randomness of natural phenomena; they are generated either by sampling uncertainty or measurement.

#### 4.2.3. Results Using Moment Matching

**Figure 14:**
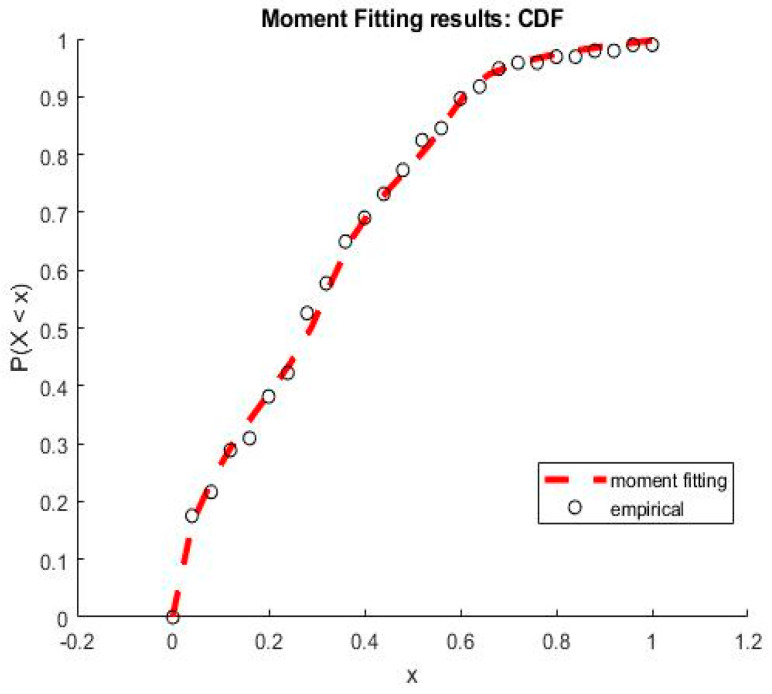
Estimation of the cumulative density function of the number of infected people by SARS-CoV-2 in Morocco. The results furnished by moment matching method.

**Figure 15:**
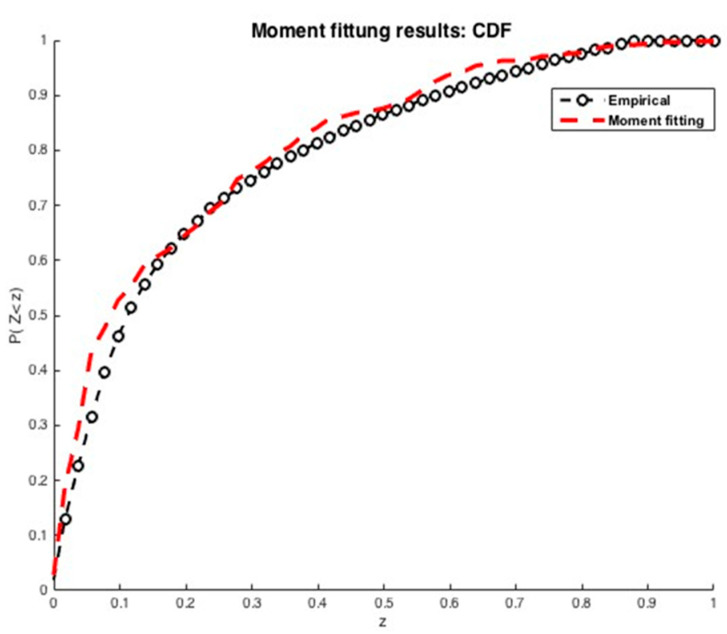
Estimation of the cumulative density function of the number of deaths by SARS-CoV-2 in Morocco. The results furnished by moment matching method.

As mentioned previously, the second alternative approach used for determining the coefficients of the expansion is moment matching. The corresponding results are exhibited in Figure 14 and Figure 15.

The results presented in Figure 1 show a good quality of approximation of the cumulative density function of the number of infected individuals by SARS-CoV-2 for an expansion of degree and n=9 and k=5 first empirical moments, with the polynomial basis $\varphi_{i}$ defined in Equation (15) and S∼Exp(1). From the corresponding CDF, one may obtain any desired statistical information of the state variables, such as mean value, variance or any given quantile of interest that will be used to establish preventive measures to manage the pandemic crisis, especially to anticipate the growth and timing in responding to the logistics needs of the health system.

Governmental agencies and health institutions should be prepared in advance for the control of epidemic outbreaks. This means that they should have in place robust contingency plans addressing issues such as vaccines and commodities, availability of emergency medical stocks and well-trained personnel, their appropriate deployment, the availability of different types of vehicles for the transportation of essential medical supplies, etc. Effective epidemic management requires the combination of managerial decisions, such as planning and resource allocation [49]. Our proposed model for epidemic risk provides useful insights for planning and mitigating against a potential risk in future.

However, the lack of any significant policies on disaster planning and implementation issues for the suggested control and intervention strategies remains a major gap in governmental planning. Our work emphasizes the importance of logistics decisions at every stage of disaster and risk management.

The results presented in Figure 15, show an approximation for an expansion of degree n = 5 and k=7 first empirical moments using a Gaussian polynomial—id est, $\varphi_{i}$ given by Equation (15) and S∼N(0,1). The estimated CDF of the number of deaths by SARS-CoV-2 presents a slightly worse solution, but it is still a reasonable approximation. One of the well-known drawbacks of the moment matching approach is that the quality of the approximation depends on the quality of the optimization—if the algorithm converges to a non-interesting global optimum of A, a poor quality of approximation is obtained. This kind of approximation is connected to Levy’s theorem [36]. Such a theorem ensures the convergence in distribution, which is a weak convergence involving the approximation of the cumulative distribution function of the variable, but not the approximation of the variables themselves [28]. In practice, it means that moment matching is not anticipated to furnish a good approximation of the variables, but only of their distributions. The coefficients of orthogonal projection ***PZ*** are given in Table 8. 

Now, we present some comparative analysis between the two proposed methods: collocation and moment matching for SARS-CoV-2 case study. We investigate the performance of the two numerical methods for the determination of the coefficients of expansion presented in Section 4: COL and MM. In the two approaches, we employed different values of ns in order to analyze its influence on the results. In the COL and MM approaches, we generate the results of this example considering the two indicators of SARS-CoV-2. In order to quantify the accuracy of each approach, we evaluate the RMSE of the approximation given by each method considering $\alpha_{i}=10\%$. The results are summarized in Table 9.

One may easily see from these results that COL provided precise results compared to MM. For the two approaches, the accuracy of the estimated values of the state variables increases as the sample size ns augments. Finally, it must be pointed out that the MM approach takes more computational time to furnish the results.

#### 4.2.4. Forecast for the Pandemic Parameters

Here, we perform predictions for the pandemic parameters (from 02 March 2020 to 20 December 2021) for the case of Morocco using the collocation approach. Most importantly, with the model and parameters in hand, we can carry out simulations and forecast the potential tendency of the COVID-19 pandemic. For that, we firstly perform the predictions of the cumulative number of confirmed cases, suspected cases and the number of deaths cases for the same period. Secondly, we investigate the degree of dependency between these key variables by computing the correlation coefficient ρ, as well as the performance of the prediction, by computing the RMSE between the model predictions and the observations.

In the following, the effect of SARS-CoV-2 is analyzed considering three state variables: susceptible (S), infected (I) and removed (R) people. “S” is used to represent the number of individuals not yet infected with the disease at time t, or those susceptible to the disease. “I” denotes the number of individuals who have been infected with the disease and are capable of spreading the disease to those in the susceptible category. “R” are those individuals who have been infected and then removed due to death [46]. The predictions of these state variables are given bellow.

**Figure 16:**
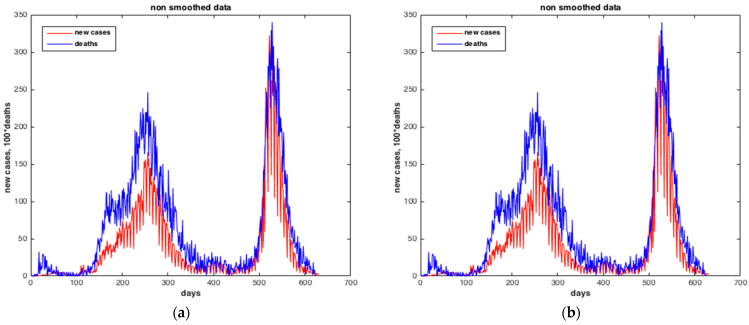
Comparison between the number of confirmed cases and the number of deaths. (**a**) Non-smoothed data. (**b**) Smoothed data. The results furnished by collocation method.

**Figure 17:**
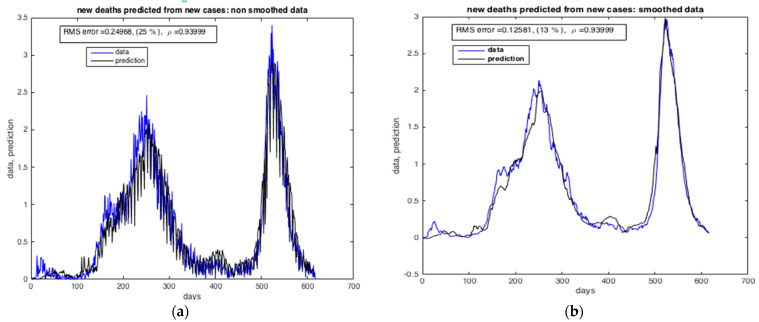
Estimation of the number of deaths by SARS-CoV-2 in Morocco predicted from the number of infected cases. (**a**) Non-smoothed data. (**b**) Smoothed data. The results furnished by collocation method.

Figure 16 and Figure 17 present the results of the estimation of the two state variables of the number of infected cases and death cases using the collocation approach. We may see from these figures that the collocation approach was able to estimate “I” and “R” with precision, i.e., with very low RMS values. The number of deaths in the blue curve reached the peak in Δ wave (from 5 July 2021 to 14 November 2021), this can be justified by the fact that Δ wave is more severe than α wave (first wave of the pandemic in Morocco).

**Figure 18:**
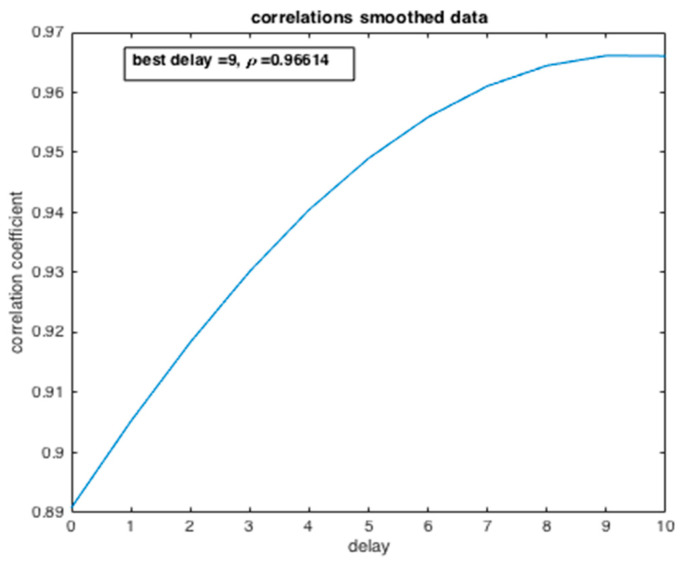
Evolution of the correlation coefficient of the number of confirmed cases (I) and the number of deaths (R).

From Figure 18, the dependency degree between the two variables, the number of infected people and the number of deaths, is examined by computing the correlation coefficient ρ. We can deduce that there is a strong dependence between the two state variables ρ=0.96 with the best delay of 9 days. Such dependency has a causal meaning, which may affect the efficiency of the preparedness strategy of the government and health system confronted with such an epidemic crisis. 

**Figure 19:**
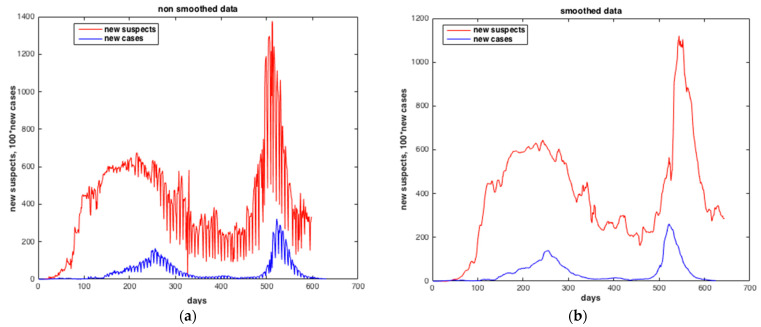
Comparison between the number of suspected cases and the number of infected cases. (**a**) Non-smoothed data. (**b**) Smoothed data. The results furnished by collocation method.

**Figure 20:**
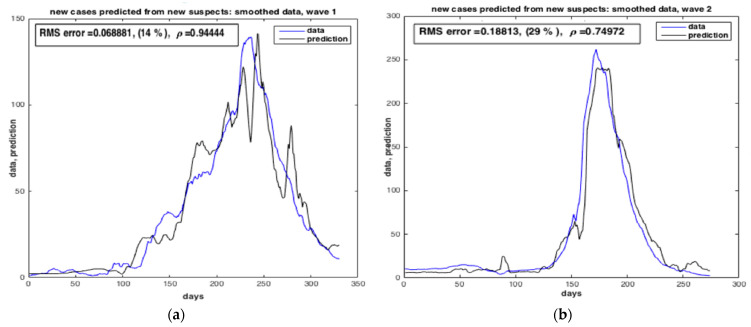
Estimation of the number of infected cases by SARS-CoV-2 in Morocco predicted from the number of suspected cases. (**a**) Case of α wave. (**b**) Case of Δ wave. The results furnished by COL.

Figure 19 and Figure 20 show the results of the estimation of the two state variables of the number of suspected cases and infected cases using collocation approach. The results indicate a good quality of estimation of “S” and “I”, i.e., with very low RMS value. The number of infected peoples in blue curve reached the peak in **Δ** wave (from 05 July 2021 to 14 November 2021).

**Figure 21:**
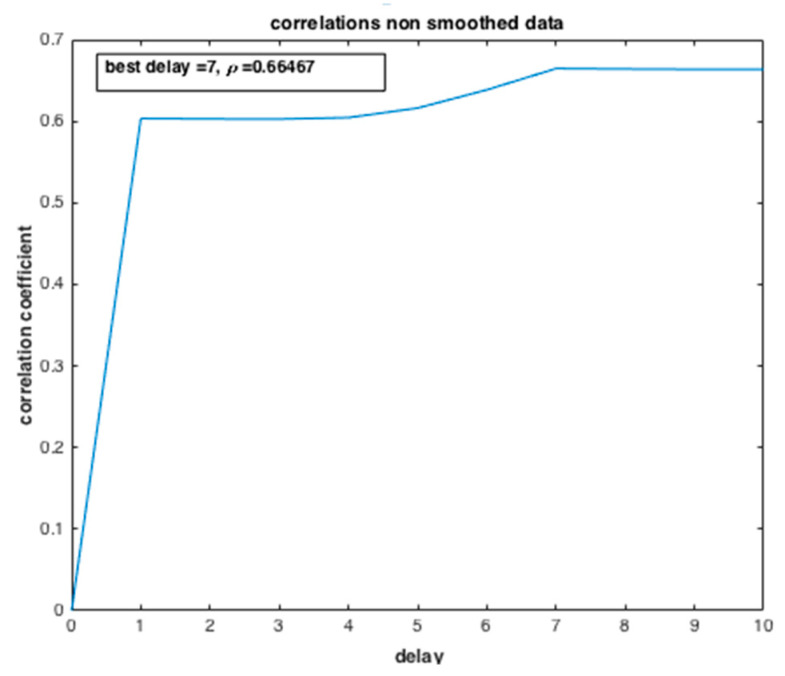
Evolution of the correlation coefficient of the number of suspected cases (S) and the number of infected cases (I).

Figure 21 investigates the dependency degree between the two variables: the number of suspected individuals, “S”, and the number of infected ones, “I”, by computing the correlation coefficient ρ. The results show low dependence between the two state variables ρ=0.66 when compared with the correlation between “I” and “R”. Such dependency is not causal, i.e., in the broadest sense, “correlation” may indicate any type of association; here it refers to the degree to which a pair of variables are linearly related.

#### 4.2.5. Probabilities of the Epidemic Disaster Impact

The interest of this work is not only looking for the cumulative distribution functions (CDFs), but also to describe the impacts of SARS-CoV-2’s behavior according to the new infected cases and the new deaths detections. For that, a classification of the disaster impact was established according to the classification of the “National Risk Assessments: A Cross Country Perspective” (OCDE, 2018) [50] for disaster evaluation.

The probabilities corresponding to Table 10 and Table 11 are computed and summarized in Table 12 and Table 13.

According to the probabilities, it is clear that SARS-CoV-2 has a sever human impact. In terms of disaster management, especially analyzing a disaster using the severity of human impacts, the epidemic risk is evidently considered as a critical disaster. 

The probabilities obtained may be used to perform predictions of the tendency of a pandemic in a region, in general, and the prediction of potential epidemic risk in future. In fact, the principle is simple; it is about making a draw with replacement of a region by taking into consideration the basic probabilities obtained by our model. 

This method is very effective and is applicable if the considered regions are independent in terms of risk/disaster occurrence (the independence of the regions may be tested by the conditional expectation). However, this method has limitations because it gives a good result only for long-term predictions. The results of this study show that SARS-CoV-2 does not have a known usual distribution and may even be a mixture of probability distributions, as shown by the cumulative distribution function (CDFs). This is justified by the fact that epidemic disasters behave randomly according to the climate seasonality, epidemiologic and clinical parameters, population behaviors and their adherence to health system instructions. Our application goes beyond the use of uncertainty quantification tools to determine the distribution of the data. It aims to use the proposed model to predict behavior and effectively manage potential epidemic risks in future. The proposed approach shows its effectiveness as an important decision-making tool for government and organizations in terms of disaster management, especially pre-disaster preparedness.

## 5. Proposed Quantitative Epidemic Risk Management Approach

**Figure 22:**
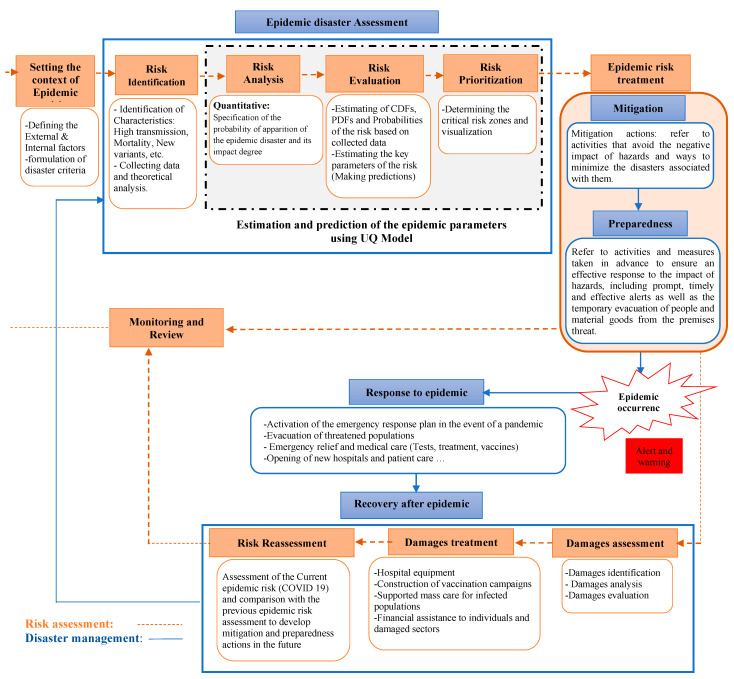
Epidemic risk management approach based on uncertainty quantification model. Estimation of epidemic parameters can be performed by collocation and/or moment matching method.

In this section, the aim is to propose a new approach for epidemic risk management based on mathematical modeling. Therefore, we move towards the concept of humanitarian logistics, a branch of logistics which deals with disaster preparedness and intervention (including epidemics). The proposed approach, shown in Figure 22, presents the interactive process of risk management deployed in disaster situations to support decision-making during each phase of the disaster management cycle.

Taking a decision at the right time during each phase leads to better preparation, better alerts, reduced vulnerability and/or prevention of future disasters. The cycle in orange as seen in the framework represents the risk management process while the blue one represents the correspondence between the stages of risk management and those of the disaster management cycle.

### 5.1. Risk Management Process

Risk management consists of identifying, evaluating and controlling the disasters to which we are exposed. The process of risk management in a disaster context contains seven basic steps [28]:

(a) Setting the context: any process of risk management begins by establishing the context of what needs to be managed. At this step we define the internal and external context, the objectives and the criteria of the epidemic disaster (rate of transmission, mortality, mutation, contamination conditions, treatment, vaccination, etc.). Setting the context helps to identify the parameters for the next steps of risk management process.
(b) Disaster risk identification: risk identification allows decision-makers to be aware of events likely to create uncertainty. This step requires an effort to analyze and identify the sources of epidemic risk.
(c) Disaster risk analysis: once the specific epidemic risk has been identified, we determine its likelihood of occurrence and its impacts. The objective of the analysis is to better understand the disaster, and how it might affect individuals and ordinary life.
(d) Disaster risk evaluation: the risk assessment is carried out using the mathematical model in order to estimate the key parameters of the epidemic, the cumulative density function and to make predictions to anticipate the behavior of the disaster, and therefore, to better propose the mitigation measures.
(e) Disaster risk prioritization: at this stage we can decide if the risk is acceptable, if it requires treatment or if it must be eliminated.
(f) Disaster risk treatment: this facilitates the elimination of the risk or reduces it to an acceptable level. We have to accept the risk, which is the retention strategy adopted by an organization, when the impact of the occurrence of an adverse event is considered to be low. However, there are cases in which taking on the epidemic disaster is not a choice, in particular in the case where the risk treatment cost is very high. The risk treatment can be matched with the response step in the disaster management cycle, where preventive and/or corrective plans to minimize the negative impacts of the disaster are implemented.
(g) Monitoring and review: this step is about continuously monitoring the new and existing epidemic risks together with the corresponding plans so that the risk management process will be reviewed and updated regarding the epidemic mutation.

### 5.2. Disaster Management Process

Disaster management includes the activities of preparation, response, improvement and reconstruction; it is a part of what is called humanitarian logistics, which consists of bringing the right material to the right place by the right person in the right quantity and the right quality, at the right time, in the case of an emergency [49,51]. Disaster management encompasses all the activities and processes designed to be implemented before, at the time of, and after the disaster, with the aim of preventing or mitigating human and material damage [52]. So, the main objective of disaster management is to reduce or avoid possible damages from disasters, ensure good help to the people in need, and plan for a quick and efficient rehabilitation and recovery [53]. Disaster management emphasizes the coordination of efforts and the management of resources and interventions to deal with all aspects of emergencies, in all aspects of mitigation, preparedness, response and recovery stages. We define five steps of the disaster management cycle [49,51]:

a. Disaster assessment: it is a set of activities to analyze and evaluate previous disasters in order to define their criticality.
b. Mitigation: this step includes laws and mechanisms that reduce the vulnerability of the population and increase their resilience.
c. Preparedness: this step refers to the implementation of response plans in order to act against factors that society has failed to avoid or mitigate.
d. Response: is the act of attending to the disaster.
e. Rehabilitation/Recovery: it is the final step of the cycle which comes after the intervention against the disaster, with the objective of restoring the normal functioning of systems and victim lives.

## 6. Conclusions and Future Works

In this paper a new approach for epidemic risk modeling based on uncertainty quantification (UQ) is presented in order to effectively predict and manage the disaster in the preparedness and response phases. This approach is based on the representation of random variables using polynomials. It consists in expanding the state variables using deterministic coefficients of this expansion. For the determination of the coefficients of the expansion, we presented two approaches: moment matching (MM) and collocation (COL). It is important to mention that the proposed approach provides not only an estimation of the state variables, but also gives their full probabilistic description (CDFs). 

In the numerical analysis section, the case of SARS-CoV-2 in Morocco, as a case of pandemic risk, was analyzed using COL and the MM method. The advantages and issues of each approach were pointed out. For instance, collocation was the more precise approach; however, it required some dependence between the variables. On the other hand, MM provided a reasonable estimation, but it required the solution of a complex optimization problem for the determination of the coefficients. More generally, the proposed epidemic risk model based on UQ was able to estimate the values of the main key parameters of SARS-CoV-2 with precision, i.e., with very low RMS values.

From the point of view of quantitative risk management, the contribution of this work is in line with considering the variable of death number, confirmed cases and suspected cases in epidemic risk, for modelling and making predictions for future behavior of the disaster under uncertainties. In fact, previous studies generally focused on risk occurrence predictions and clinical views, and they did not take into consideration uncertainties for disaster modeling. The available data themselves are uncertain due to the fraction of non-detected cases infected by SARS-CoV-2, so in this work, this kind of uncertainty was taken into consideration in the model validation. In general, the method has shown its effectiveness and given us a good quality of results which we used to calculate the probabilities of the risk impacts and thus to propose a new approach of risk management based on quantitative indicators of potential epidemic disasters. 

This standard method for the knowledge and assessment of disasters is a fundamental element for the strengthening of risk management policies in Morocco and more in general worldwide. Its implementation will significantly improve the effectiveness of plans, whether for risk prevention, crisis management preparation, or post-disaster recovery and reconstruction, which are the subject of future works. It will be based on the use of optimization tools for facility location problems. In addition, other mathematical models will be developed for the prediction of natural and technological disasters, in particular Markov chains and neural networks. Therefore, a comparison will be made between the models for the final validation of the developed pre-disaster approach. 

## Figures and Tables

**Table 1:** Data used in the first example.

$S_{1}$	−1.237	−0.938	−0.810	−0.760	−0.694	0.0256	0.215	0.308	1.281	2.011
$S_{2}$	0.0477	0.212	0.290	0.386	0.604	0.615	0.6445	1.1215	2.119	3.309

**Table 2:** Results furnished by COL in the first example.

	On the Data	On the Uniform Grid	On the Random Grid
RMSE	4 × 10^−16^	7 × 10^−16^	9 × 10^−5^
Relative error	3 × 10^−16^	2 × 10^−16^	8 × 10^−5^

**Table 3:** Coefficients found in the second example by COL.

	$z_{1}$	$z_{2}$	$z_{3}$	$z_{4}$	$z_{5}$	$z_{6}$	$z_{7}$
N(0,1)	−0.019	0.0211	−0.0793	−0.1576	0.0484	−0.1444	0.0561
Exp(1)	0.041	−0.0505	0.2561	−0.5479	0.5250	−0.1851	0.6812

**Table 4:** Coefficients found in the first example.

	$z_{1}$	$z_{2}$	$z_{3}$	$z_{4}$	$z_{5}$	$z_{6}$
$Z_{1}$	−2.3268	−2.2921	57.8965	−151.6174	167.4003	−62.8043
$Z_{2}$	−7.7497	56.4476	−142.3897	103.5209	84.0354	−85.5012

**Table 5:** Coefficients found in the second example by MM.

	$z_{1}$	$z_{2}$	$z_{3}$	$z_{4}$	$z_{5}$	$z_{6}$
N(0,1)	−0.2610	1.4849	−3.7193	5.7942	−3.9630	0.9815

**Table 6:** Morocco summary statistics per day.

Values	Total Cases	New Cases	Total Deaths	New Deaths	Total Tests	Total Vaccinations	People Vaccinated	People Fully Vaccinated
mean	6.07 × 10^5^	1402.74	9180.02	18.22	5.27 × 10^6^	2.37 × 10^7^	1.34 × 10^7^	1.13 × 10^7^
std	4.39 × 10^5^	2072.41	5969.70	24.81	3.57 × 10^6^	1.81 × 10^7^	8.11 × 10^6^	7.98 × 10^6^
min	1	0	1	0	2.9 × 10^1^	0.0 × 10^0^	2.36 × 10^6^	4.87 × 10^3^
25%	1.57 × 10^5^	135	2976	2	2.09 × 10^6^	8.61 × 10^6^	5.04 × 10^6^	4.26 × 10^6^
50%	5.18 × 10^5^	473	9143	6	5.31 × 10^6^	1.92 × 10^7^	1.08 × 10^7^	9.45 × 10^6^
75%	9.84 × 10^5^	1848	14,904	28	8.61 × 10^6^	3.93 × 10^7^	2.32 × 10^7^	2.07 × 10^7^
max	1.26 × 10^6^	12,039	16,269	127	1.17 × 10^7^	5.52 × 10^7^	2.49 × 10^7^	2.35 × 10^7^

**Table 7:** Estimation of Polynomial function of key parameters of SARS-CoV-2 using COL.

Parameters	Polynomial Approximation Using Exponential Random Variable S					
New infected cases by SARS-CoV-2	$z_{1}$	$z_{2}$	$z_{3}$	$z_{4}$	$z_{5}$	$z_{6}$
	0.9912	−23.46	816.56	−1360.03	961.34	−276.13
New deaths by SARS-CoV-2	$z_{1}$	$z_{2}$	$z_{3}$	$z_{4}$	$z_{5}$	$z_{6}$
	0.0156	−0.6938	20.30	−54.42	59.07	12.85

**Table 8:** Estimation of polynomial function of key parameters of epidemic risk using MM.

Parameters	Polynomial Approximation Using Exponential Random Variable S									
New infected cases by SARS-CoV-2	$z_{1}$	$z_{2}$	$z_{3}$	$z_{4}$	$z_{5}$	$z_{6}$	$z_{7}$	$z_{8}$	$z_{9}$	$z_{10}$
	0.002	−0.19	3.49	6.54	−174.94	949.91	−2461.81	3350.45	−2305.13	632.72
New deaths by SARS-CoV-2	$z_{1}$	$z_{2}$	$z_{3}$	$z_{4}$	$z_{5}$	$z_{6}$				
	0.0027	0.0986	0.6217	−1.5966	1.7479	0.7187				

**Table 9:** RMSE of the state variables of SARS-CoV-2 for $\alpha_{i}=10\%$.

		Number of Infected by SARS-CoV-2		Number of Deaths by SARS-CoV-2	
Approach	ns	RMSE	Time (s)	RMSE	Time (s)
MM	100	$1.66\times 10^{-3}$	600	$1.44\times 10^{-3}$	560
	500	$1.02\times 10^{-4}$	780	$5.05\times 10^{-4}$	690
	1000	$2.50\times 10^{-5}$	1200	$3.15\times 10^{-5}$	1080
COL	100	$5.34\times 10^{-1}$	0.27	$8.07\times 10^{-1}$	0.30
	500	$2.87\times 10^{-2}$	0.33	$6.62\times 10^{-2}$	0.32
	1000	$1.85\times 10^{-3}$	0.36	$5.24\times 10^{-3}$	0.40

**Table 10:** Classification of the human impact of SARS-CoV-2 based on new cases detection.

Classes	Human Impact
Low	1 to 9 new cases per day
Medium	10 to 49 new cases per day
Major	More that 50 new cases per day

**Table 11:** Classification of the human impact of SARS-CoV-2 based on new deaths detection.

Classes	Human Impact
Low	1 to 4 deaths detected per day
Medium	5 to 19 deaths detected per day
Major	More than 20 deaths detected per day

**Table 12:** New cases classes probabilities. Results obtained by collocation.

New Cases Classes	Related Probabilities	Probabilities with Uncertainties
Low	0.00915	0.00847
Medium	0.00449	0.00453
Major	0.98636	0.987

**Table 13:** New deaths classes probabilities. Results obtained by collocation.

New Deaths Classes	Related Probabilities	Probabilities with Uncertainties
Low	0.00509	0
Medium	0.35051	0.29093
Major	0.6444	0.70907

## Data Availability

The data collected was taken from official sources, the information relating to is detailed in the references attached to the manuscript.
